# *Mycobacterium tuberculosis* Infection Induces BCSFB Disruption but No BBB Disruption In Vivo: Implications in the Pathophysiology of Tuberculous Meningitis

**DOI:** 10.3390/ijms23126436

**Published:** 2022-06-09

**Authors:** Carlos Sánchez-Garibay, Citlaltepetl Salinas-Lara, Marcos Artemio Gómez-López, Luis O. Soto-Rojas, Nidia Karen Castillón-Benavides, Omar Jorge Castillón-Benavides, María Elena Hernández-Campos, Rogelio Hernández-Pando, Brenda Marquina-Castillo, Manuel Alejandro Flores-Barrada, José Alberto Choreño-Parra, Juan Carlos León-Contreras, Martha Lilia Tena-Suck, Dulce Adriana Mata-Espinosa, Porfirio Nava, Jessica Medina-Mendoza, Cesar Augusto Rodríguez-Balderas

**Affiliations:** 1 Departamento de Neuropatología, Instituto Nacional de Neurología y Neurocirugía Manuel Velasco Suárez, Mexico City 14269, Mexico; carlos.s.garibay@live.com.mx (C.S.-G.); mltenasuck@gmail.com (M.L.T.-S.); 2 Red MEDICI, Carrera Médico Cirujano, Facultad de Estudios Superiores Iztacala, Universidad Nacional Autónoma de México, Tlalnepantla 54090, Mexico; oskarsoto123@unam.mx (L.O.S.-R.); choreprr@gmail.com (J.A.C.-P.); jesse_2291@hotmail.com (J.M.-M.); 3 Laboratorio de Patogenesis Molecular, Laboratorio 4, Edificio A4, Carrera Médico Cirujano, Facultad de Estudios Superiores Iztacala, Universidad Nacional Autónoma de México, Tlalnepantla 54090, Mexico; 4 Instituto Nacional de Rehabilitación (INR), “Luis Guillermo Ibarra Ibarra”, Mexico City 14389, Mexico; golma76@gmail.com; 5 Unidad de Especialidades Médicas de la Secretaria de la Defensa Nacional, Mexico City 11200, Mexico; bena4vides@gmail.com; 6 Neurological Center, American British Cowdry Hospital, Mexico City 05330, Mexico; castillon_omarjorge@hotmail.com; 7 Sección de Estudios de Posgrado e Investigación, Escuela Superior de Medicina del Instituto Politécnico Nacional, Plan de San Luis y Díaz Mirón s/n, Casco de Santo Tomás, Mexico City 11340, Mexico; mayehc@hotmail.com; 8 Experimental Pathology Section, Department of Pathology, National Institute of Medical Science and Nutrition “Salvador Zubirán”, Mexico City 14080, Mexico; rhdezpando@hotmail.com (R.H.-P.); dulmat@yahoo.com.mx (D.A.M.-E.); 9 Department of Pathology, National Institute of Medical Science and Nutrition “Salvador Zubirán”, Mexico City 14080, Mexico; brenda.marquinac@incmnzs.mx; 10 División Académica Multidisciplinaria de Comalcalco de la Universidad Juárez Autónoma de Tabasco, Comalcalco 86658, Mexico; manbarsito@gmail.com; 11 Laboratorio de Inmunobiología y Genética, Instituto Nacional de Enfermedades Respiratorias Ismael Cosío Villegas, Mexico City 14080, Mexico; 12 Tecnologico de Monterrey, Escuela de Medicina y Ciencias de la Salud, Mexico City 14380, Mexico; 13 Laboratorio de Microscopia Electrónica, Departamento de Patología, Instituto Nacional de Ciencias Médicas y Nutrición Salvador Zubirán, Mexico City 14080, Mexico; carlos.leonc@incmnsz.mx; 14 Department of Physiology, Biophysics and Neuroscience, Mexico City 07360, Mexico; pnava@fisio.cinvestav.mx; 15 Servicio de Pediatría, Hospital Juarez de México, Secretaria de Salud, Mexico City 07760, Mexico; 16 Departamento de Bioterio, Instituto Nacional de Neurología y Neurocirugía Manuel Velasco Suárez, Mexico City 14269, Mexico; neurovet.eeg@gmail.com

**Keywords:** tuberculosis, central nervous system, meningitis, blood-brain barrier, blood-cerebrospinal fluid barrier, choroid plexus, neuro-inflammation

## Abstract

Central nervous system (CNS) tuberculosis is the most lethal and devastating form among the diseases caused by *Mycobacterium tuberculosis*. The mechanisms by which *M. tuberculosis* bacilli enter the CNS are still unclear. However, the BBB and the BCSFB have been proposed as possible routes of access into the brain. We previously reported that certain strains of *M. tuberculosis* possess an enhanced ability to cause secondary CNS infection in a mouse model of progressive pulmonary tuberculosis. Here, we evaluated the morphostructural and molecular integrity of CNS barriers. For this purpose, we analyzed through transmission electron microscopy the ultrastructure of brain parenchymal microvessels and choroid plexus epithelium from animals infected with two mycobacterial strains. Additionally, we determined the expression of junctional proteins and cytokines by immunological techniques. The results showed that the presence of *M. tuberculosis* induced disruption of the BCSFB but no disruption of the BBB, and that the severity of such damage was related to the strain used, suggesting that variations in the ability to cause CNS disease among distinct strains of bacteria may also be linked to their capacity to cause direct or indirect disruption of these barriers. Understanding the pathophysiological mechanisms involved in CNS tuberculosis may facilitate the establishment of new biomarkers and therapeutic targets.

## 1. Introduction

Until the coronavirus pandemic, tuberculosis (TB) was the leading cause of morbidity and mortality worldwide attributed to a single infectious agent, ranking above HIV/AIDS. The World Health Organization (WHO) estimates that this bacillus caused 10 million new infections and 1.5 million deaths in 2020, with most of the cases occurring in low-income countries [1]. One of the most devastating forms of tuberculosis is meningitis, due to its high mortality and the sequels it leaves on survivals, which reflects the difficulty in diagnosing and delay in initiating treatment [2]. Central nervous system(CNS) tuberculosis could also occur as tuberculoma, tuberculous abscess tuberculous encephalopathy, Pott’s spine, Pott’s paraplegia, non-osseous spinal tuberculoma, spinal meningitis, and vasculitis with secondary hemorrhage or infarction, and most of these forms share an intense inflammatory response as a common feature [3]. However, the bacillus reaches the brain and remains silent within nervous tissue for some time before the affected individual manifests symptoms.

Currently, the way by which the pathogen enters the brain has yet to be completely elucidated. The CNS is separated from the systemic circulation by the blood–brain barrier (BBB) and blood–cerebrospinal-fluid barrier (BCSFB), the former constituted by endothelial cells supported on a basement membrane and surrounded by pericytes and the end foot processes of astrocytes, whereas the latter is formed by a layer of epithelial cells connected by cell junctions at the choroid plexus [4,5]. These barriers limit the access of circulating substances or infectious agents to the CNS, due to the characteristics of endothelial cells that make them capable of exerting selective permeability, as well as preventing paracellular transport because of the tight junctions that hold them together [6]. Nevertheless, some pathogens are causative of neuroinfection because their virulence factors allowing them to adhere to these cells and cross such barriers [7,8], whereas in the specific case of *Mycobacterium tuberculosis* (*M. tuberculosis*), there are no sufficient studies that evaluate the mechanisms contributing to the crossing of brain barriers and the colonization of the CNS by this bacillus.

Some hypotheses have been proposed to explain how this bacterium breaches BBB. On the one hand, *M. tuberculosis* may access the CNS inside an infected monocyte [9]; on the other, it may cross as an extracellular organism. It has also been observed that certain cytokines could disrupt endothelial and epithelial barriers altering the expression of junctional proteins [10,11], but the role of cytokines in the breakdown of brain barriers and the penetration of *M. tuberculosis* into the CNS remains to be elucidated. Additionally, studies published so far have not evaluated the role of structural alteration of the BSCFB and BBB in facilitating access into the CNS, since research has previously focused on the behavior during infection [12].

We previously reported that certain strains of this genus, isolated from patients with tuberculous meningitis, caused dissemination and brain infection in a mouse model of progressive pulmonary tuberculosis induced by intratracheal inoculation [13]. Therefore, the aims of this study were to confirm that some genotypes of *M. tuberculosis* have an enhanced ability to cause damage to the CNS, and to evaluate structural changes and junctional protein expression in the BBB and BCSFB during the inflammatory response induced by experimental pulmonary tuberculosis, using a strain isolated from a different patient with meningeal tuberculosis.

## 2. Results

### 2.1. Survival Rate, Lung Histopathology, and Pulmonary Bacterial Load

Strains showed varying virulence. The rate of mortality was significantly higher for strain N15 than that exhibited by H37Rv, particularly at the finale of the experiment. The group infected with the H37Rv strain had a 95% survival rate at week 17 post-infection; at the same point, the N15 infected group had a 5% survival rate. Survival curves were analyzed with Kaplan–Meier plots and Logrank tests.

Bacterial load was not detected from pulmonary tissue of infected mice with any strains in the early days post-infection. Bacterial growth was detected until 14 days post-infection (DPI) in both infected groups. At 14 and 21 DPI, the bacterial load per gram of pulmonary tissue of the N15 strain was significantly lower than that of H37Rv, while at 28 and 60 DPI, no significant difference was detected between the bacterial loads of the two infected animal groups.

**Figure 1:**
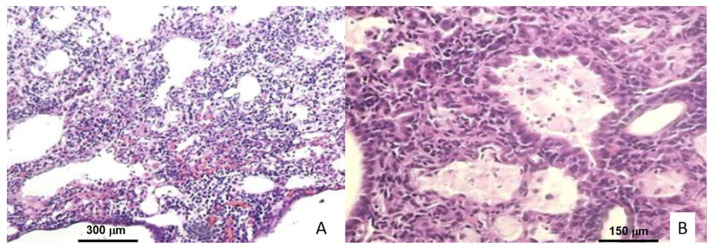
Histological micrographs of Balb/C mice lung after 28 days of infection with *M. tuberculosis* H37Rv. (**A**) Extensive inflammatory infiltrates, granulomas, and pneumonic areas (40× magnification). (**B**) Foamy and epithelioid cells (400× magnification).

Microscopic analysis of lung tissue from mice infected with both strains showed that on days 1, 3, and 7, the alveolar lumen was clean, with preserved histological architecture and patent bronchioles, distinctive characteristics of healthy tissue. In the experimental subjects sacrificed on day 14, the tissue architecture was observed intact; however, congestive blood vessels and few peribronchial lymphocytes were seen. From day 21, granulomas and pneumonia were observed in the tissues, with the progression of the affected area determining the day of sacrifice. However, the extension of the lesions was vaster in the tissues of animals infected with the N15 strain (Figure 1). The acid-fast bacilli (AFB) were detected in the central structure of granulomas and inside free macrophages.

### 2.2. Encephalon Histopathology and Bacterial Loads

Tissue obtained from animals infected with any of the two strains showed no bacterial growth in culture media. However, the IS6110 sequence was amplified in the cerebral hemispheres of mice inoculated with N15 at 3, 7, 21, and 28 DPI. PCR was not performed for H37Rv, as it was previously demonstrated that this strain has a low ability to colonize the CNS [12].

**Figure 2:**
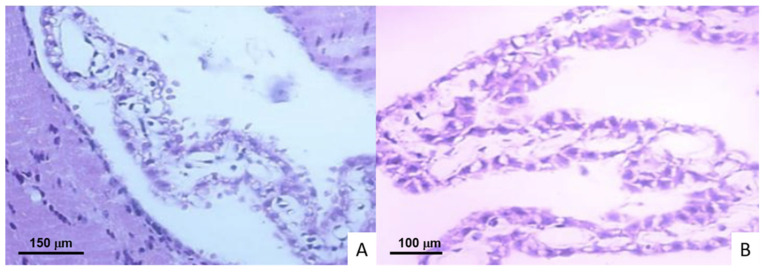
Histological micrographs of Balb/C mice choroid plexus. (**A**) 28 and (**B**) 60 DPI with *M. tuberculosis* H37Rv ependymal cells with inflammatory characteristics, cytoplasmic vacuolation, and architectural distortion (400× and 600× magnification, respectively).

Histopathological analysis revealed atypical neurons with eosinophilia, loss of neuron polarity, alteration of the organization of neuronal layers, necrosis, and interstitial and cytotoxic edema in the cortex and hippocampus in the two groups of infected animals. At 1, 3, and 7 DPI, no changes were observed in the choroidal plexuses of mice infected with the *M. tuberculosis* H37Rv strain. Among the cytopathic effects caused by the bacilli were inflammatory atypia and vacuolization in the ependymal cells at 14 and 21 DPI. Loss of architecture and inflammatory infiltration manifested at 28 DPI. In addition to these, changes in nuclear polarity and cytoplasmatic retraction were present at 60 and 120 DPI (Figure 2).

The brain cortex of mice infected with the H37Rv showed scarcer neurons with cytoplasmic retraction at 1 and 3 DPI. These increases in cytopathic effects continued until 21 DPI, adding to the degeneration of neurons, and ependymal reaction was observed. On the same day, alterations in polarity and in the hyperchromatic nuclei were detected in the frontal cortex, and from 60 DPI, pronounced stratification loss and axonal cone collapse, along with major alterations in the polarity of the neurons, were determined in the tissue sections. In the hippocampus, histopathological changes were observed at 14 DPI in the forms of hyperchromatic nuclei and inconspicuous and ependymal reactions. At 60 DPI, neuronal decline, eccentric nuclei, and karyorrhexis were detected in CA1 and CA3 areas.

For the mice infected with N15 strain, from 1 DPI, cytoplasmic retraction and hyperchromatic nuclei were detected in the brain cortex, while at 3 DPI, added focal neuronal degeneration, stratification loss and axonal cone collapse, major alterations in polarity, and loss of neuropil were observed in the frontal cortex, changes that increased with the progression of the experiment. The hippocampal region on 1 and 3 DPI exhibited the same alterations described in the brain cortex; these increased until 14 DPI. At 21 DPI, neurons showed karyorrhexis and important signs of degeneration. Neuronal decline in the CA3 area and loss of pyramidal neurons were detected in 28 DPI. Besides these alterations, at 60 DPI, eccentric nuclei, karyolysis, and neuronal decline in the CA1 area were additionally noted.

From 1 and 3 DPI, cytopathic effects caused by N15 (LAM 3) strain were documented in choroidal plexuses, cytoplasmic vacuolization, edema, and inflammatory atypia in ependymal cells. At 7 and 14 DPI, an increase in intercellular spaces and vacuolization was observed in almost all cells of the plexuses. Microvilli and polarity loss were observed at 21 DPI, with a progressive increase in cytoplasmic vacuolization until the end of the experiment.

**Figure 3:**
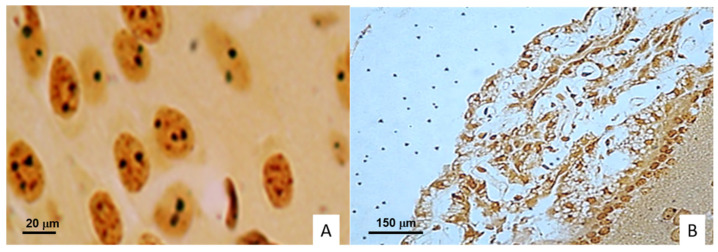
Histological micrographs of encephalon from Balb/C mice infected with *M. tuberculosis* N15 (LAM3) strain. (**A**) Brain cortex neurons with nuclei retraction, loss of chromatin, nucleolus preservation, and evident AgNORs (400× magnification). (**B**) Ependymal cells of choroid plexus with inflammatory characteristics, cytoplasmic vacuolation, and architectural distortion. Bielschowsky stain at 40× magnification.

In the argyrophilic nucleolar organizer region (AgNOR) slides, reduction in nuclear area was detected from 7 DPI, as well as karyolysis with preservations of nucleoli and NORs, increasing until the last DPI (Figure 3). However, histopathological-alteration AFB were not detected by Ziehl Neelsen.

### 2.3. Mycobacterium tuberculosis Infection Induces Disruption of BCSFB but Not of BBB at the Ultrastructural Level

Although histopathological images did not show differences in the morphological changes induced by both strains of *M. tuberculosis* in the BBB and BCSFB, our analysis of ultrastructural architecture by transmission electron microscopy (TEM) revealed that N15 caused severe damage from the onset of disease compared with H37Rv.

**Figure 4:**
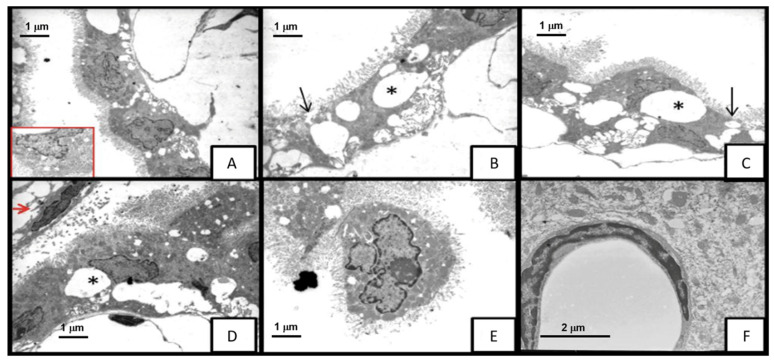
Electron micrograph showing the morphological changes in mice infected with *M. tuberculosis* H37Rv strain at 21DPI. (**A**–**D**) Choroid plexus cells with intracytoplasmic vacuolation *, cell membrane alterations and tight junction separation (arrows), (**D**) presence of Kolmer or epiplexus cells (red arrow) ×8000. (**E**) The ependymal cell completely separated from the others, with shortening of the microvilli ×8000. (**F**) Hippocampal blood vessel showing endothelial cell without apparent alterations ×10,000.

Choroid plexus epithelial cells from animals infected with H37Rv presented the following cytopathic effects: discrete cytoplasmic vacuolization at 14 DPI; formation of large lacunar spaces by the vacuoles on day 21, some cells showing loss of classical cuboidal shape with flattening of microvilli, and the space between them increasing, with some vacuoles occupying 50% of the cell (Figure 4). Additionally, we observed detachment of cells from the basement membrane at 28 and 60 DPI, and mitochondrial swelling at 120 DPI.

**Figure 5:**
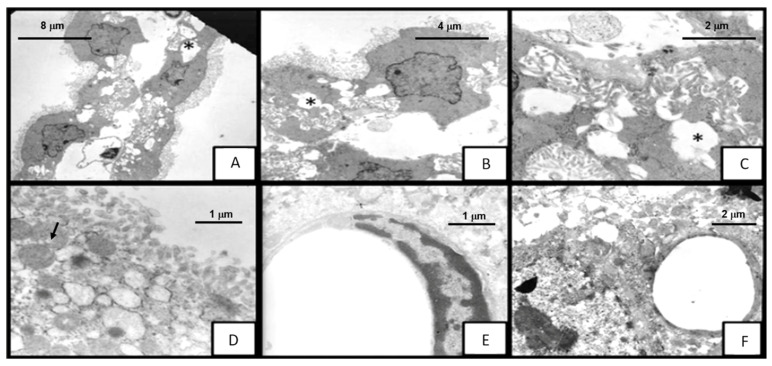
Transmission electron micrograph of brain tissue of mice infected with *M. tuberculosis* N15 (LAM 3) strain on day 3: the epithelial cells of the choroid plexus with evident alteration of the architecture; vacuolation of the more than 60% of the cell area; enlargement of the interdigitations and separation of the cells *. (**A**) ×2500, (**B**) ×5000, (**C**) ×10,000 (**D**) observed with increased vacuolation and mitochondria alteration (black arrow) ×12,500. (**E**) Frontal cortex blood vessel and ×12,500; (**F**) hippocampus blood vessel ×8000, without alterations of the architecture.

On the other hand, N15 caused almost the same alterations on the choroid plexus, but these appeared earlier and were more severe in the degree of cytoplasmic vacuolization (Figure 5A–E). In fact, vacuoles could be observed from 3 DPI occupying more than 60% of the cellular area, accompanied by increased intercellular space within the epithelium, mitochondrial enhancement with blurred cristae, and microvilli flattening. At 7 DPI, there was a detachment of cells from the basement membrane with necrosis; at day 14, loss of cytoplasm, nude nuclei, organelle degranulation, and leucocyte infiltration (mononuclear and polymorphonuclear cells) were found. Vacuolization occupied 90% of the cellular area at 60 DPI, whereas the separation between cells as well as detachment from the basement membrane and mitochondrial changes peaked on day 120.

Conversely, we did not observe any loss of architecture in the neurovascular unit in the hippocampal and frontal cortex parenchymal vessels of mice infected with both strains of *M. tuberculosis* (Figure 5E,F).

### 2.4. Profiles of Cytokine Expression in BCSFB during Mycobacterium tuberculosis Infection

H37Rv induced a proinflammatory profile of cytokine production, as we observed immunoreactivity to TNF-α and IL-1β within the cytoplasm of choroidal cells on the first day of pulmonary infection, which remained positive during all times of sacrifice. IL-4 and TGF-β only were expressed on the last day of the model in ependymal cells and choroid plexus.

Inoculation with the N15 strain induced an intermittent reactivity of TNF-α and IL-1β in ependymal cells, choroid plexus, and capillaries from 14 DPI to the last day of the experiment. IL-4 was detected in the same structures from 3 DPI until the end of the experiment. TGF- β was negative on all days (Figures S1 and S2).

### 2.5. Altered Expression of Junctional Proteins in BCSFB during Experimental Pulmonary Tuberculosis

**Figure 6:**
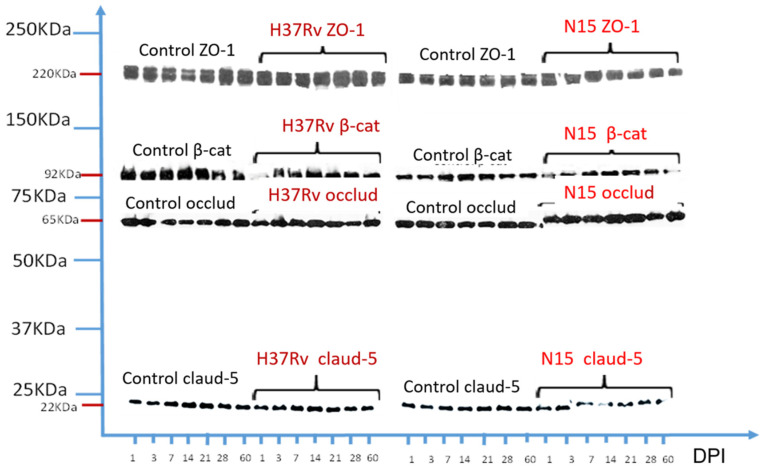
Semiquantitative western blot of changes in cell junction expression in brains of mice infected with *M. tuberculosis* strains. As we can observe, among the ZO-1 proteins, occludin was phosphorylated during the kinetics of the experiment; claudin-5, in addition to being phosphorylated, shows a decrease in its expression with the *M. tuberculosis* N15 (LAM3) strain.

It has been shown that proinflammatory cytokines disrupt different epithelial barriers [10]. Therefore, we next investigated the effect of pulmonary infection with both strains of *M. tuberculosis* on the expression of several cell junctional proteins in the CNS. For this purpose, lysates obtained from the brain tissue of mice infected with the N15 or the H37Rv strain for 1–60 days were analyzed by western blotting. As shown in Figure 6, no changes in β-catenin and occludin were detected in any condition. However, downregulation of claudin-5 and ZO-1 was observed after 7 and 21 DPI, respectively, in the N15 strain. Interestingly, the reduction in these two proteins was accompanied by an increase in occludin. No changes in any cell junction proteins were perceived in the animals exposed to the H37Rv strain. Taken together, these results suggest that the N15 strain affects the cerebral protein expression of cell junctions.

Nonetheless, brain lysates were obtained from complete hemispheres, rather than from specific cells of blood vessels or choroid plexus. We performed immunofluorescence to determine whether the observations made by immunoblotting were due to alterations of junctional proteins in the BBB or BCSFB. According to our previous results, H37Rv did not alter the expression of claudin-5, occludin, and ZO-1, nor did it change the endothelial cells of brain microvessels or the epithelial cells of choroid plexus (unpublished data).

**Figure 7:**
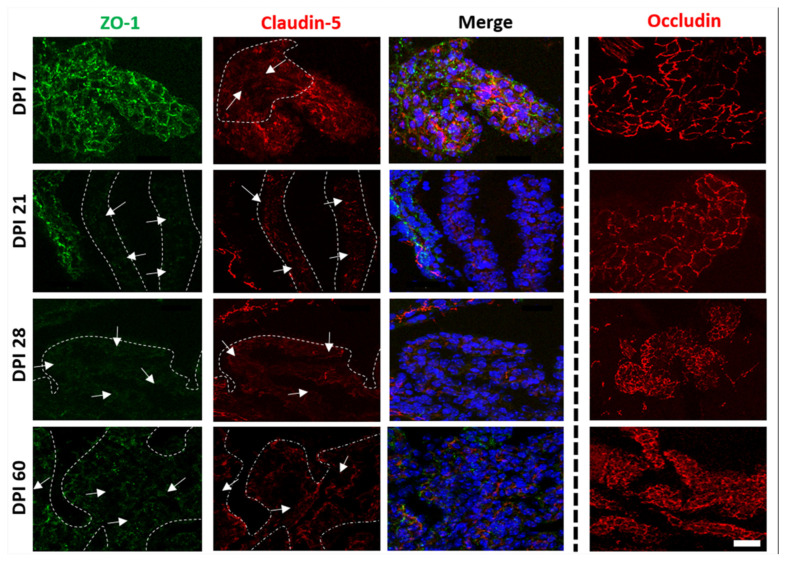
Temporal course of fluorescence immunolabeling for cell junctions in choroid plexus of mice infected with *M. tuberculosis* N15 (LAM 3) at different DPI. Arrows and dotted lines correspond to areas devoid of junctional proteins. The scale bar = 20 μm is common for all micrographs.

**Figure 8:**
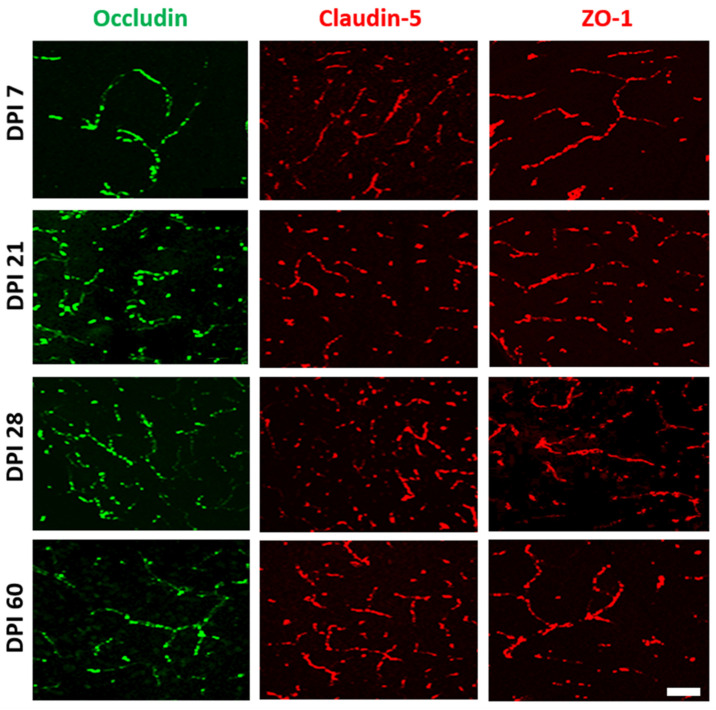
Temporal course of fluorescence immunolabeling for cell junctions in brain tissue of mice infected with *M. tuberculosis* N15 (LAM 3). Scale bar = 20 μm is common for all micrographs.

Conversely, and consistent with the results of western blot, the tissue sections from animals infected with the N15 strain showed an altered pattern, and a decreased expression of claudin-5 and ZO-1 was observed after 7 and 21 DPI, respectively, in the BCSFB (Figure 7). Such alterations consisted of diminished expression and changes in the localization of proteins within the cell. However, we observed reorganization and an augmented fluorescence signal in occludin after 60 days of exposure (Figure 7). No changes were found in the BBB (Figure 8).

## 3. Discussion

Central nervous system tuberculosis is the most devastating form of human mycobacterial infection, but at the same time, one of the least understood. Until now, some aspects, such as the mechanisms by which *M. tuberculosis* leaves the lungs and reaches the brain, have been little-known; there are several hypotheses, but only some of these have been evaluated, and only in experimental animal models [14].

In this work, under light microscopy, no histological alterations were detected in the encephalon structures of the mice inoculated with either of the *M. tuberculosis* strains. In previous work, we established a model of cerebral tuberculosis with different strains isolated from the CSF of Colombian patients with tuberculous meningitis inoculated intratracheally into BALB/c mice, imitating hematogenous dissemination from the lung, and demonstrated that certain genotypes of *M. tuberculosis* manifest differences in their ability to colonize the CNS [13]. Following the same line, in this paper, we used a strain obtained from a Mexican patient who presented with CNS tuberculosis without evidence of active disease in the lung. To our knowledge, this is the first study to evaluate the structures of the BBB and BCSFB in the context of the systemic inflammatory response induced by progressive pulmonary tuberculosis.

Be et al. (2008) reported that the intravenous inoculation of BALB/c and outbred Hartley mice with H37Rv or CDC 1551 *M. tuberculosis* strains did not generate morphological alterations in the brain and a low increase in the concentrations of TNF-α, IL-1, and e IL-10 in disrupted tissue. These results contrast with those of this work, which found alterations in the ependymal cell layer of lateral ventricles and the pia mater, including changes in the expression of IL-4 and INF-α that differed from those observed in humans. Regarding survival, in this work, all the animals started to die at 3 weeks post-infection, and 95% of all animals were dead at 17 weeks post-infection, in contrast to the animals of Be et al. (2008), in which all the mice died between the sixth and seventh week post-infection. It is important to note the difference in the infection pathway: the intravenous administration of the bacterial load in the work of Be et al. induced a higher lethality [15].

Moreover, there are animal models to study this disease, and those studies that have not used existing animal models have used the intracranial or intravenous route to inoculate the pathogen without mimicking the spread from the lung [16,17].

There are two studies in which important morphological alterations were observed; the first was by van Well et al. in 2007, in which the authors inoculated 5 × 10^5^ bacteria of the H37RV strain directly into the brains of C57B16 mice. In these animals, they observed the development of tuberculomas and the presence of inflammatory infiltrates in addition to purulent and infarcted areas. In the study by Mazzolla et al. in 2002, the brains of mice were also directly inoculated with 1 x 10^6^ CFU *M. bovis* bacillus Calmette-Guérin Montreal (BCG-M) strain. Massive infiltration of inflammatory cells into brain parenchyma was observed. The development of morphological alterations is related to the bacterial strain and the concentration of the pathogen that reaches the brain tissue [17,18].

Interestingly, the clinical strain used in this study, although isolated from a different individual, was the same genotype of strain 209 that was reported in our previous paper, which could reflect a relationship between the incidence of tuberculous meningitis with the prevalence of certain genotypes of *M. tuberculosis* within affected patients, as previously suggested [19]. Such strains are shown to be more lethal and to possess an enhanced ability to colonize the CNS, causing inflammation at different anatomical sites within the parenchyma.

Here, we confirm our previous observations: that the N15 strain has a higher lethality and its presence in the nervous system could be detected by PCR. Additionally, we observed alterations in the morphology of the choroid plexus after lung infection with this strain, as well as with H37Rv; however, no alterations were noted in the morphology of the BBB. Using TEM analysis, we found that damage to the BCSFB was more severe with N15, revealing that differences in the ability to cause CNS disease between distinct genotypes of bacteria may also be linked to their capacity to cause direct or indirect injury to the BCSFB. Such damage consisted of cellular changes suggestive of toxicity, as we observed signs of necrosis, although the specific cause of cytotoxicity could not be evaluated. One possibility is that *M. tuberculosis* may have virulence factors that allow it to mediate direct cytotoxicity against choroid plexus epithelial cells. In the lung, this cytotoxicity may be due to the ability of the different strains of *M. tuberculosis* to enter the alveolar epithelial cells and trigger an immune response. McDonough and colleagues demonstrated that H37Rv causes direct cytotoxicity against cells of lung epithelium [20]. Likewise, Rivas et al. demonstrated through in vitro assays that alveolar epithelial cells, excluding human macrophages, efficiently produce human β-defensin 2 (HBD-2) after infection with *M. tuberculosis* (H37Rv strain), suggesting that HBD-2 from alveolar epithelial cells may be a key component of the innate immune response against *M. tuberculosis* [21]. However, future experiments are required to determine and compare these pathological events with the N15 (LAM3) strain. Taken together, these mechanisms could be important for the hematogenous spreading of *M. tuberculosis* from the lung. Other authors have observed that choroid plexus epithelial cells are also susceptible to cytotoxicity mediated by direct contact with different pathogens causative of neuroinfection in a strain-dependent way [22]. Therefore, it would be of great interest to evaluate such a mechanism in subsequent works and to determine whether certain strains of *M. tuberculosis* have an enhanced capacity to induce death in BCSFB cells.

The other possibility is that certain molecules released from lung lesions induce cell death at distant sites and cause loss of brain-barrier functions. In fact, serum levels of vascular endothelial growth factor (VEGF) are increased in patients with active pulmonary tuberculosis [23]. VEGF augments permeability and disrupts several endothelial barriers, including the BBB [24], whereas its role in the loss of BCSFB properties is not clear. However, its blockade was shown to diminish fenestration and augment the thickness of endothelial cells of the choroid plexus vasculature [25]. Cytokines are alternative candidates, as they are released from the lung into the blood in patients with active pulmonary tuberculosis, and some of them alter the characteristics of several epithelial barriers [10]. The choroid plexus responds to acute [26] and chronic [27] systemic inflammatory stimuli, altering their pattern of transcription and upregulating the expression of genes involved in pathways that facilitate the entry of cells into the cerebrospinal fluid. IFN-γ regulates the entrance of inflammatory cells into the CNS by modulating the epithelial expression of trafficking molecules at the choroid plexus [28]. TNF-α disrupts the BCSFB by induction of apoptosis dependent on caspase and NFkB activation [29]. Inflammatory breakdown of the BCSFB by TNF-α is also related to increased activity of metalloproteases, augmented expression of mRNA of vascular cell adhesion molecule-1 (VCAM-1)and intracellular adhesion molecule-1 (ICAM-1), and altered distribution of tight junction proteins [30].

On the other hand, other routes of access for *M. tuberculosis* into the CNS have been suggested: (1) by non-specific mechanisms, such as the entry into the BBB or BBCB of a macrophage that has endocytosed *M. tuberculosis*, a mechanism known as “Trojan Horse” [31]; (2) by crossing the BBB through vasculitis and infecting endothelial cells [32]; (3) via ligand-receptor interaction. Regarding the latter, Be et al. reported that in the absence of the pknD protein (a serine-threonine protein kinase), it does not interfere with colonization or persistence in the CNS, but changes the adhesion of *M. tuberculosis* to the endothelial cells of the BBB. Therefore, pknD is a key element for bacterial invasion and virulence in the CNS [33]. In addition, according to the high similarity between *M. tuberculosis* and *M. leprae* [34,35], the neurotropism of *M. tuberculosis* may be due to the same adhesion factors that allow the specific interaction with the laminin-alpha2 (LN-alpha2) with *M. leprae* molecules [36].

It is interesting that although both H37Rv and N15 disrupted the choroid plexus structure and induced cytokine expression at this level, the effects triggered by infection with N15 occurred earlier and were more severe, and only this strain caused changes in the expression of junctional proteins. The above could be related to the pattern of cytokines induced by exposure to each strain. We documented in a previous paper that N15 elicits a profile of cytokines characterized by the predominance of TNF-α and IL-4, with low induction of IFN-α within the lung and brain. Here, we observed a similar pattern of cytokine expression at the choroid plexus with persistent positivity for IL-4 along the different times of sacrifice, but with intermittent reactivity to IFN-γ, TNF-α, and IL-1β. As mentioned above, proinflammatory cytokines IFN-γ and TNF-α alter the functions of the BCSFB, [30,37,38] whereas the effect of IL-4 on the maintenance of properties of such barriers has not been described. Baruch and colleagues [37] found that the decline in the IL-4: IFN-γ ratio with increased levels of IL-4 in the choroid plexus, affected function, morphology, and expression of junctional proteins at the BCSFB, suggesting that a predominance of Th2 cytokines alter epithelial-T lymphocyte crosstalk, which is detrimental to the choroidal epithelium, and that this could be another example of epithelial pathologies mediated by a Th2 inflammation, such as the lung epithelium in asthma. Even so, it is difficult to explain why H37Rv did not modify the expression of junctional proteins, since this strain induced a profile of cytokines with a proinflammatory effect that, as mentioned before, alters the properties of the BCSFB. In this regard, a limitation of our study is that we did not analyze such proteins from cells specific to the choroid plexus, and instead of doing so, we performed western blotting from lysates of complete hemispheres. Despite this, the immunofluorescence assay revealed differences between the two genotypes of *M. tuberculosis* in their capacity for modifying the expression of such proteins.

In addition, our results strongly suggest that although H37Rv showed minimal brain infection, it is not completely harmless to the CNS. The above is reinforced by the fact that, in addition to the alterations found in the choroid plexus, we also observed inflammatory changes within the cerebral parenchyma induced by lung infection with H37Rv, which had not previously been described. This phenomenon may be explained at least by two possibilities. First, it could be related to the entry of blood-borne cytokines into the brain, which could modify CNS function and induce inflammation [38,39]. In our previous paper, although H37Rv could not be isolated from brain tissue, it induced mild proinflammatory cytokine expression [13]. On the other hand, antigens of H37Rv may gain entry into the brain and activate local immune responses without the need for the presence of living bacteria in the tissue. In fact, we have observed that subcutaneous inoculation of antigens from heat-killed bacilli causes inflammatory infiltrates in the brain parenchyma (unpublished data), although we have not defined whether such antigens enter the CNS via the BBB or BCSFB, or whether they are sufficient to alter the function and structure of such barriers.

Finally, despite our results supporting previous research speculating on the possible involvement of the choroid plexus in the pathophysiology of cerebral tuberculosis and raising the BCSFB as a new site to which to direct our attention [40], in the model reported here, we did not demonstrate which of these was the site of entry used by *M. tuberculosis* to gain access into the CNS. Although we could not observe disruption of the BBB, we cannot rule out entrance through this barrier, since previous data from in vitro models indicate that the bacillus may cross endothelial cells without altering the integrity of monolayers [11] and we observed bacteria inside the cytoplasm of endothelial cells in our previous article [13].

In conclusion, we demonstrated here for the first time that pulmonary infection with certain strains of *M. tuberculosis* can induce changes in brain barriers and can specifically alter the integrity of the BCSFB, which may be related to the ability of some genotypes of bacteria to colonize the brain. However, the mechanisms involved in the induction of such damage and the importance of this phenomenon for the pathophysiology remain to be cleared. This denotes and reinforces the fact that the interaction between *M. tuberculosis* and the host is complex and multifaceted, and that the type of immune response generated will trigger the outcome of the infection.

## 4. Materials and Methods

### 4.1. Experimental Animals

Male BALB/c mice, 6–8 weeks old, were infected and maintained in specific pathogen-free conditions in a Biosafety Level 3 animal facility in the National Institute of Medical Science and Nutrition “Salvador Zubirán”.

### 4.2. Mycobacterial Strains

*M. tuberculosis* N15 is a clinical strain isolated from the cerebrospinal fluid of a Mexican patient with tuberculous meningitis. This was genotyped as a Euro-American strain of the Latin American Mediterranean, LAM-3, ST-33, with a spoligotyping pattern: 776177607760771, the same reported to 209 from (HP-2010 y RB 2021), and the MIRU-VNTR designation: 122116133212. *M. tuberculosis* H37Rv obtained from ATCC was used as a control.

Bacterial strains were grown in liquid medium Middlebrook 7H9 (DifcoTM Middlebrook 7H9 Broth) supplemented with glycerol, sodium chloride, bovine albumin, dextrose, and catalase (BBLTM Middlebrook ADC Enrichment), incubated with shaking at 100 rpm and 37 °C over 21 days. Growth was monitored by densitometry until the culture reached the mid-log phase, a density of 600nm. Then, bacilli were harvested and suspended in phosphate-buffered saline (PBS) containing 0.05% Tween 80, and shaken for 10 min with 2-millimeter diameter glass beads to disperse bacterial clumps. The suspension was centrifuged for 1 min at 350× *g* to remove the remnant clumps. Aliquots were stored at −70 °C until use, and random samples were used to perform bacterial count by the determination of colony-forming units (CFUs). Aliquots were diluted to achieve aliquots of 2.5 × 10^5^ CFU per 100 μL of PBS as infective inoculum, CytoSoft Data Acquisition and Analysis Software, version 4.2.1 (Guava Technologies, Inc., Hayward, CA, USA).

### 4.3. Experimental Model of Progressive Pulmonary Tuberculosis

Mice were anesthetized with sevoflurane and infected with 2.5 × 10^5^ CFU per 100 μL of PBS via intratracheal injection as previously described [41]. Animals were separated into three groups; one infected with H37Rv, the other with N15, and a control group to which PBS was administrated. Sacrifice was performed at 1, 3, 7, 14, 21, 60, and 120 days post-infection (DPI), and nine animals per group were killed at each date. Four mice were killed by exsanguination and five mice were anesthetized with sodium pentobarbital, and cardiac perfusion with 4% paraformaldehyde in Sorensen’s phosphate buffer 0.2 M pH 7.2 by a peristaltic pump was then performed. Lungs and brains from perfused mice were dehydrated, embedded in paraffin, and used for histologic examination, Ziehl-Neelsen staining, immunohistochemistry (IHC), and TEM, whereas brains from unperfused animals were frozen at −80 °C and used later to determine CFUs and for analysis of junctional proteins by western blotting and immunofluorescence.

### 4.4. Preparation of Tissue Sections for Histological Analysis, Ziehl-Neelsen Staining, and Immunohistochemistry

Formalin-fixed paraffin-embedded tissue was sectioned with a microtome to obtain sections of 2 μm. Slides were dyed with hematoxylin-eosin, Pio del Rio Hortega staining technique, AgNOR, and Ziehl-Neelsen for histological analysis and determination of acid-fast bacilli. For immunohistochemistry, sagittal brain sections were used.

Standard immunohistochemical (IHQ) staining protocol was performed using primary antibodies against TNF-α (TNF-alpha Antibody (68B6A3 L1), Invitrogen; Waltham, MA, USA), IFN-γ (sc-373727 Santa Cruz Biotechnology, Inc. IL-1β (IL-1β Antibody (H-153), Santa Cruz Biotechnology, Inc.; Dallas, TX, USA), IL-4 (IL-4 Antibody (C-19), Santa Cruz Biotechnology, Inc.) and TGF-β (TGFbeta RI (T-19), Santa Cruz Biotechnology, Inc.). A Mouse/Rabbit ImmunoDetector DAB HRP Detection System was used for primary antibodies detection (BioSB; Goleta, CA, USA) and tissues were counterstained with hematoxylin. At least three slides from two different specimens were analyzed with a Carl Zeiss Primo Star Image Analyzer with an integrated Zeiss Axiocam ERc camera (White Plains, NY, USA).

### 4.5. Determination of Mycobacterium tuberculosis CNS Infection by Colony-Forming Units (CFUs)

A cerebral hemisphere of each animal per day of sacrifice was set in a cryotube with 800 µL of PBS and an approximate volume of 200 µL glass beads, and homogenized with a mini bead beater. Four decimal serial dilutions of each homogenate were spread onto duplicate plates containing Bacto Middlebrook 7H10 agar (Difco™). After 14 and 21 days, CFUs were counted and adjusted per gram of tissue. Infection was confirmed by the amplification of insertion sequence IS6110 by conventional PCR as described before [42].

### 4.6. Transmission Electron Microscopy (TEM)

Formalin-fixed paraffin-embedded tissues were deparaffinized with xylol, rehydrated, fixed with glutaraldehyde in cacodylate buffer (pH 7.2), and postfixed with 1% osmium tetroxide. Then, tissue was dehydrated via ethanol series, infiltrated with propylene oxide, and embedded in Epon resin. Ultrathin sections cut with ultramicrotome were stained with Toluidine blue, counter-stained with uranyl acetate, and examined using a Carl Zeiss EM10 transmission electron microscope.

### 4.7. Determination of Junctional Proteins in Endothelial Cells of the BBB and Epithelial Cells of BCSFB

Junctional proteins claudin-5, occludin, ZO-1, and intracellular signal transducer β-catenin were determined by western blotting. For this purpose, frozen brain tissue was homogenized as described before for CFUs. Then, the sample was treated with Triton X-100 and centrifuged to obtain the non-precipitated fraction with the protein extract. Equal amounts of protein lysates were separated by SDS-polyacrylamide gel electrophoresis (SDS-PAGE) and were transferred onto a nitrocellulose membrane by electroblotting (110V, 60min, 4 °C). Next, we blocked unspecific binding sites with TBS containing 5% skim milk for 1 h, and then added primary antibodies. After overnight incubation, the samples were washed three times in TBS containing 0.05% Tween-20, probed with species-specific peroxidase-conjugated secondary antibodies for 1 h, and revealed with SuperSignal™ West Pico Chemiluminescent Substrate (Thermo-Fisher Scientific, Waltham, MA, USA). Chemiluminescence signals were recorded on a ChemiDoc imaging device (Biorad, Mexico-City, Mexico).

Claudin-5, occludin, and ZO-1 were also assessed by immunofluorescence in parenchymal blood vessels and choroid plexus epithelial cells. Briefly, brain tissue was embedded in the Tissue-Tek O.C.T. compound and sectioned with a cryotome at −20 °C. Sections 30 µm thick were blocked with a solution of 1% bovine serum albumin (BSA) in PBS for 20 min at room temperature. Subsequently, they were washed and incubated with primary antibodies at 4 °C overnight, and with secondary antibodies—FITC-tagged goat-anti-rabbit IgG secondary antibody and TRITC-tagged goat anti-Mouse IgGγ secondary antibody (Jackson Immuno Research Laboratories, Inc., West Grove, PA, USA)—for 1 h, then counterstained with carbocyanine monomer nucleic acid stain (TO-PROR-3 iodide, Thermo Fisher Scientific) and mounted with anti-quenching media (Vectashield Antifade Mounting Media, Vector Laboratories, Burlingame, CA, USA). All sections were viewed through a confocal laser scanning microscope (TCS-SP2, Leica, Heidelberg, Germany).

## Data Availability

The data that support the findings of this study are available from the corresponding author upon reasonable request.

## Supplementary Materials

The following supporting information can be downloaded at: https://www.mdpi.com/article/10.3390/ijms23126436/s1.
