# Supplementary material for: Mycobacterium tuberculosis Infection Induces BCSFB Disruption but No BBB Disruption In Vivo: Implications in the Pathophysiology of Tuberculous Meningitis
Source: Int J Mol Sci. 2022 Jun 9;23(12):6436. doi: 10.3390/ijms23126436 (PMC9223849; doi:10.3390/ijms23126436)
Supplement: Supplementary file 1 [file ijms-23-06436-s001.zip › ijms-1759353-supplementary.pdf]

Supporting Information

Mycobacterium tuberculosis Infection Induces BCSFB Disruption but No BBB Disruption In Vivo: Implications in the Pathophysiology of Tuberculous Meningitis

Carlos Sánchez-Garibay, Citlaltepetl Salinas-Lara*,†, Marcos Artemio Gómez-López, Luis O. Soto-Rojas, Nidia Karen Castillón-Benavides, Omar Jorge Castillón-Benavides, María Elena Hernández-Campos, Rogelio Hernández-Pando, Brenda Marquina-Castillo, Manuel Alejandro Flores-Barrada, José Alberto Choreño-Parra, Juan Carlos León-Contreras, Martha Lilia Tena-Suck, Dulce Adriana Mata-Espinosa, Porfirio Nava, Jessica Medina-Mendoza and Cesar Augusto Rodríguez-Balderas.

* Correspondence: cisala69@hotmail.com; Tel.: +52-55-5606-3822.
† Current address: Neuropathology Department, National Institute of Neurology and Neurosurgery "Manuel Velasco Suárez", Insurgentes Sur Ave., #3877 La Fama, Mexico City 14269, Mexico.

Table of Contents

1. Cytopathic effects and immunoreactivity in ependymal cells caused by *M. tuberculosis* N15………………..S2
2. Cytopathic effects and immunoreactivity in ependymal cells caused by *M. tuberculosis* H37RV…………....S3

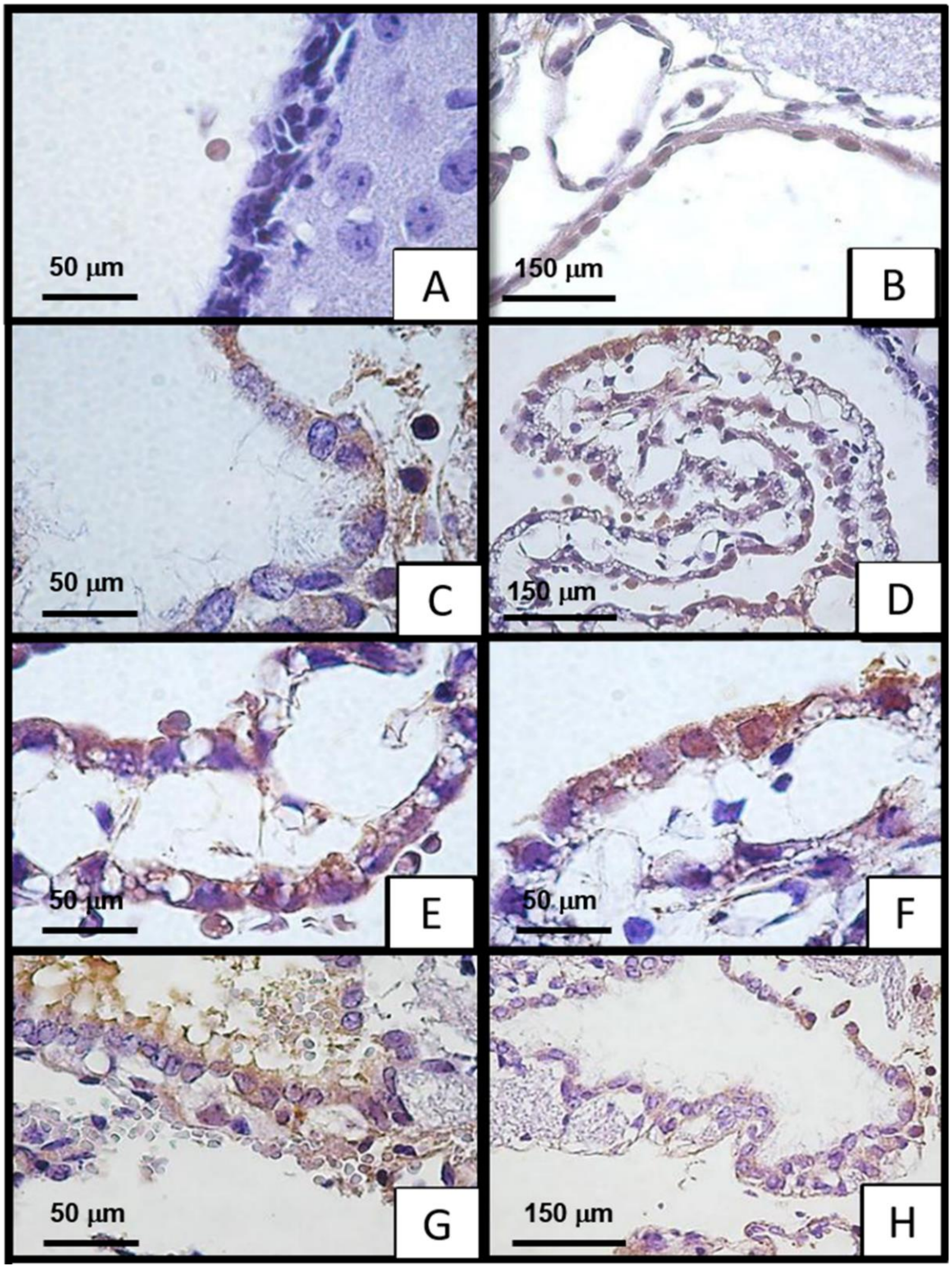

Figure S1. Histological micrographs of ependymal cells from Balb/C mice infected with *M. tuberculosis* LAM3/N15 immunoreactive to different cytokines by IHC. (A) Hyperchromatic, cytoplasmatic retraction, and pseudostratification of ependymal cells from the lateral ventricle (1000X). (B) Piamadre, IFN-γ at 60 DPI (400X). (C) Detached ependymal cells, IFN-γ positive with loss of organization at 60 DPI (1000X). (D) Choroid plexus, with loss of organization TNF-α at 120 DPI (400X). (E) Choroid plexus, with degeneration and vacuolization. TNF-α at 120 DPI (1000X). (F) Detached ependymal cells, TNF-α positive with loss of organization at 120 DPI(1000X). (G) Detached ependymal cells, IFN-γ positive with loss of organization at 120 DPI (1000X). (H) Brain cortex endothelial cells are positive for TNF-α with loss of organization and degenerative changes (400X).

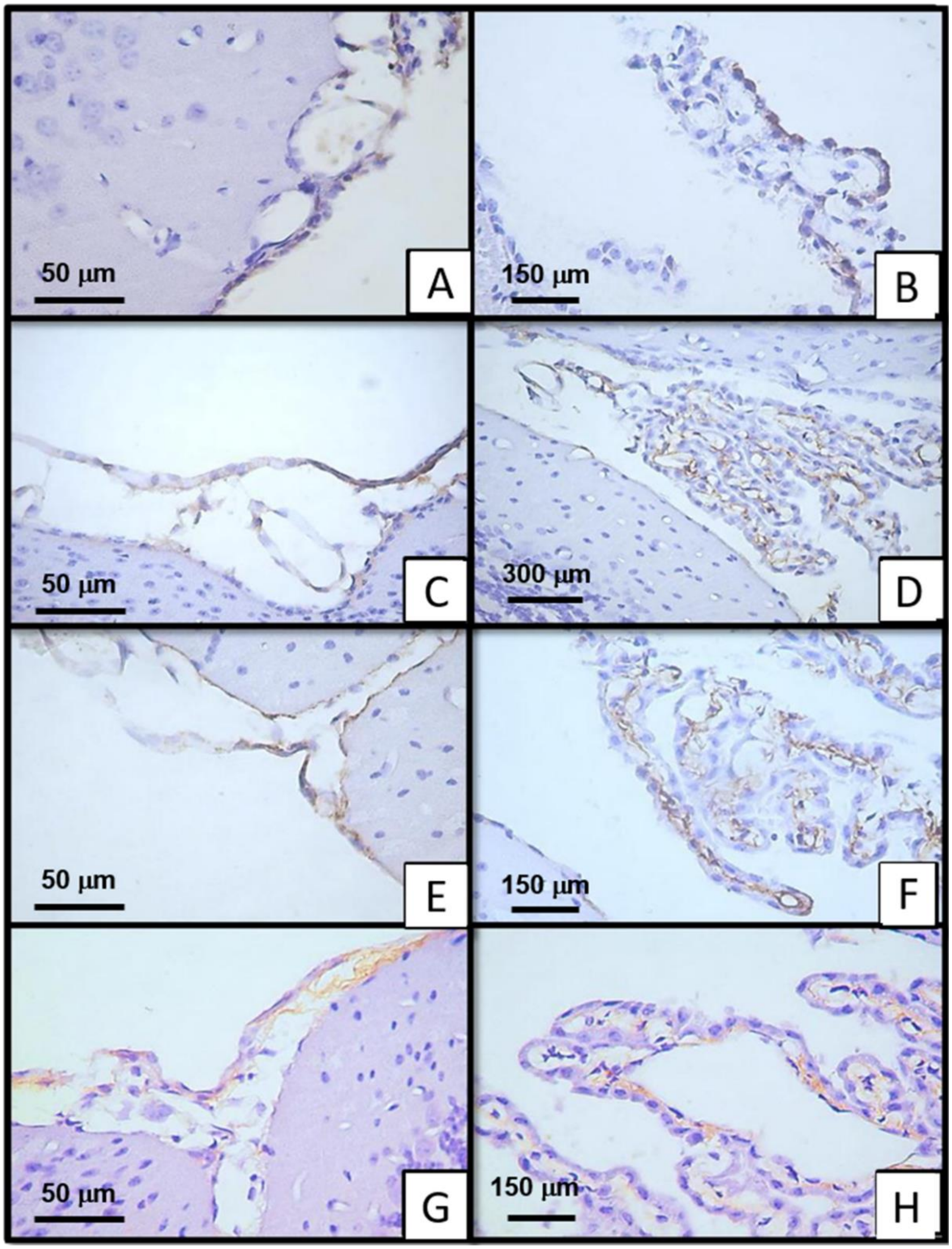

Figure S2. Histological micrographs of ependymal cells from Balb/C mice infected with *M. tuberculosis* H37Rv are immunoreactive to the presence of different cytokines by IHC. (A) Brain cortex, TNF-α at 28 DPI (400X). (B) Choroid plexus, TNF-α at 60 DPI (100X). (C) Brain cortex, IL-1β at 60 DPI. (D) Choroid plexus, IL-1β at 120 DPI (40X). (E) Brain cortex, IL-4 at 120 DPI. (F) Choroid plexus, IL-4 at 120 DPI (100X). (G) Brain cortex, TGF-β at 120 DPI. (H) Choroid plexus, TGF-β at 120 DPI (100X).
